# Transcriptome Analysis Reveals Differences in Key Genes and Pathways Regulating Carbon and Nitrogen Metabolism in Cotton Genotypes under N Starvation and Resupply

**DOI:** 10.3390/ijms21041500

**Published:** 2020-02-22

**Authors:** Asif Iqbal, Qiang Dong, Xiangru Wang, Huiping Gui, Hengheng Zhang, Xiling Zhang, Meizhen Song

**Affiliations:** 1 State Key Laboratory of Cotton Biology, Institute of Cotton Research, Chinese Academy of Agricultural Sciences, Anyang 455000, China; asif173aup@gmail.com (A.I.); dongqiang@caas.cn (Q.D.);; 2 School of Agricultural Sciences, Zhengzhou University, Zhengzhou 450000, China

**Keywords:** cotton, nitrogen, starvation, resupplying, RNA-sequence, coexpression network analysis, metabolism

## Abstract

Nitrogen (N) is the most important limiting factor for cotton production worldwide. Genotype-dependent ability to cope with N shortage has been only partially explored in cotton, and in this context, the comparison of molecular responses of cotton genotypes with different nitrogen use efficiency (NUE) is of particular interest to dissect the key molecular mechanisms underlying NUE. In this study, we employed Illumina RNA-Sequencing to determine the genotypic difference in transcriptome profile using two cotton genotypes differing in NUE (CCRI-69, N-efficient, and XLZ-30, N-inefficient) under N starvation and resupply treatments. The results showed that a large genetic variation existed in differentially expressed genes (DEGs) related to amino acid, carbon, and nitrogen metabolism between CCRI-69 and XLZ-30. Further analysis of metabolic changes in cotton genotypes under N resupply showed that nitrogen metabolism and aromatic amino acid metabolism pathways were mainly enriched in CCRI-69 by regulating carbon metabolism pathways such as starch and sucrose metabolism, glycolysis/gluconeogenesis, and pentose phosphate pathway. Additionally, we performed an expression network analysis of genes related to amino acid, carbon, and nitrogen metabolism. In total, 75 and 33 genes were identified as hub genes in shoots and roots of cotton genotypes, respectively. In summary, the identified hub genes may provide new insights into coordinating carbon and nitrogen metabolism and improving NUE in cotton.

## 1. Introduction

Nitrogen (N) is one of the most important and limiting factors for plant growth and productivity [1] and serves as a constituent of many important macromolecules, including proteins, enzymes, metabolites, signaling compounds, and several plant hormones [2]. The application of N fertilizer has significantly increased crop production and as a result, reduced the pressure of population growth [1]. However, low N application is one of the major problems in crop production and decreases the yield up to 50% [3,4]. Thus a large amount of N fertilizers is being used to improve growth and productivity [5,6], and the application may increase by threefold in the future [7] due to increasingly-declined soil fertility and widely planted high-yield crop cultivars. The increase in N fertilizer not only increases the cost of production but also brings environmental problems. In addition, it is well documented that only less than half of the applied N fertilizer is used by crops [8], with the surplus contributing to severe environmental pollution, such as groundwater contamination and soil acidification [9]. In addition, the intensive use of N fertilizers significantly increases production costs [10]. In order to reduce this costly component of crop production, there is an immediate need to reduce N fertilizer inputs. To compensate for that reduction, improved crop genotypes must be sought with higher nitrogen use efficiency (NUE) [11,12,13], as it is a basic and also the most efficient approach for coping with low N availability in the soil and insufficient N fertilizer supply. It has been well documented that NUE is a genetically controlled trait, differing dramatically among genotypes, such as in Arabidopsis [14], as well as in crops including wheat [15], rice [16], maize [17], barley [18], and rapeseed [19]. However, N-efficient cotton cultivars have not yet been cultivated specifically in China due to the lack of potential cultivars [20]. Meanwhile, we have identified some cotton genotypes with contrasting NUE in our previous studies [9,20]. Therefore, it may be assumed that N-efficient cotton genotypes contain some unique mechanisms of nitrogen and carbon metabolism.

There is still a lack of information on the correlation between N assimilation and its ability to improve carbon metabolism in plants [21]. The acquisition of N was closely linked with photosynthetic activity and carbon status of the plant in a complex regulated system known as carbon–nitrogen balance [21,22]. The carbon and nitrogen balance is very important for optimum plant growth and development [23]. Studies have shown that the well-coordinated metabolic pathways for carbon and nitrogen metabolism have a great role in determining plant growth and productivity [19,24]. Other research indicated that overexpressing the genes responsible for nitrogen metabolism had altered plant growth and development, yield, carbon, and nitrogen metabolism [25]. Moreover, the balance between carbon and nitrogen is very important for improving NUE, as sufficient carbon needs to be available for enhancing plant’s ability to uptake and utilize nitrogen. Nitrogen levels in plants can significantly affect carbon fixation [26], as N is present in large quantities in photosynthetic proteins, such as Rubisco and PEP carboxylase. Decreases in N assimilation and storage will thus decrease the overall amount of carbon fixed by the plant [27]. In addition to the C-to-N ratio, the products of glutamine synthetase (GS) and glutamate synthase (GOGAT) pathway are also crucial for plants. Glutamate acts as a signaling and N transport molecule and is also a substrate in the production of other amino acids [27,28], and glutamate also regulates carbon and nitrogen metabolism in plants. Increased expression of the enzyme PEP carboxylase has shown to result in increased levels of glutamine [28]. The changes that occur in source or sink concentrations, of either carbon or nitrogen, must be coordinated with changing environmental inputs, such as nitrogen availability [22]. While regulation of C-to-N ratios have shown to be crucial for proper plant growth and development, it also serves as a limitation when evaluating how to increase NUE in crop plants [29]. Nitrogen uptake and assimilation as well as remobilization is in part regulated and controlled by carbon metabolism, thus leading to a plateau in NUE unless the carbon metabolism is also increased.

In addition, extensive transcriptomic studies have investigated nitrogen metabolisms in plants at various levels, such as time points after N treatments [30], N sources [31], and rates [32], in cell [33], and tissue types [34]. Many genes and regulatory networks have been identified, which are involved in nitrogen metabolism [35,36]. For instance, nitrate application to N-starved Arabidopsis markedly alters the expression levels of genes responsible for primary and secondary metabolism, cellular growth, hormone responses, protein synthesis, signal transduction, and transcriptional regulation [37]. In another study, more than 1000 genes changed with the application of nitrate in Arabidopsis seedlings [38]. Similarly, in rice roots, the expression level of hundreds of genes was changed when the N supply was increased from 0 to 0.5 mM within 3 h [39]. Hundreds of genes were differentially expressed when nitrate was supplied to N-starved tomato plants [40]. Moreover, the application of nitrate to N-starved tomato plants significantly increased the transcripts levels of genes responsible for nitrate transport, nitrate reductase, and nitrite reductase in the first 24 h, but diminished after 48 h [40]. Keeping in view these studies, significant progress has been done in the identification of the networks affected by nitrate treatments in plants, however, no single study has been done to identify the molecular networks of carbon and nitrogen metabolism in cotton, which might be the base for high NUE. 

Cotton (*Gossypium* L.), known as white gold, is the backbone of the world’s natural textile fibers and is grown all over the world in more than 50 countries [41]. However, the limited availability of soil nitrogen and the lower NUE of cotton are two important factors that have seriously affected cotton production in recent years [9,20]. In our previous experiments, we evaluated 100 cotton genotypes for NUE and related traits at the seedling stage, and based on the results, these cultivars were divided into N-efficient and N-inefficient genotypes [20]. Further, six contrasting N-efficient cotton genotypes were grown in hydroponic culture, and the results showed large genotypic differences in N metabolizing enzymatic activities, dry matter weights, and NUE related traits, etc., which should be the focus of subsequent research [9]. However, to date, no study has been done to analyze the genetic difference of transcriptomic profiles in response to N starvation and resupply. It is imperative for us to reveal the underlying mechanism or to explore the relevant genes and pathways related to carbon and nitrogen metabolism, which may be responsible for high NUE in cotton genotypes. Thus, understanding the molecular mechanism of carbon and nitrogen metabolism, as well as metabolic regulatory networks, will help to improve the NUE in cotton. Therefore, we investigated the genetic networks and pathways involved in carbon and nitrogen metabolism in two cotton genotypes (CCRI-69, N-efficient, and XLZ-30, N-inefficient) in response to nitrogen resupply after a period of five days N starvation using RNA-Seq. 

## 2. Results

### 2.1. Summary of RNA Sequencing Results

In RNA-Seq, the number of clean reads was 29,285,040, 22,859,989, 23,222,951, 23,584,486, 22,206,156, 21,816,409, 22,737,237, and 23,703,549 in T1, T2, T3, T4, T5, T6, T7, and T8, respectively, after trimming and filtering (Table 1). An average (based on three biological replicates) 5.99 gigabases (14×) of clean reads (Q30 > 94.34%) was obtained in these eight samples. The frequency of a >30 Phred quality score (Q30) was > 95.22% and the guanine-cytosine (GC) content > 44.88% for the eight samples. These findings attest to the fine quality of the sequencing results. The respective mapped reads information between root and shoot of XLZ-30-0h was: 91.37% and 96.38% total mapped; 4.31% and 4.96% multiple mapped; 87.10% and 91.42% uniquely mapped, respectively. Between root and shoot of XLZ-30-6h samples, 91.28% and 94.60% were total mapped, and 4.41% and 4.84% were multiple mapped, while 86.87% and 89.75% were uniquely mapped. Similarly, the respective mapped reads information between root and shoot of CCRI-69-0h was 93.23% and 91.41% total mapped; 4.43% and 5.03% multiple mapped; and 88.80% and 86.55% uniquely mapped, respectively. Between root and shoot of CCRI-69-6h samples 89.45% and 94.88% were total mapped, and 4.86% and 4.83% were multiple mapped, while 84.79% and 90.05% were uniquely mapped (Table 1). The transcriptome data were deemed suitable for subsequent analysis.

### 2.2. Differentially Expressed Gene Analysis

**Figure 1:**
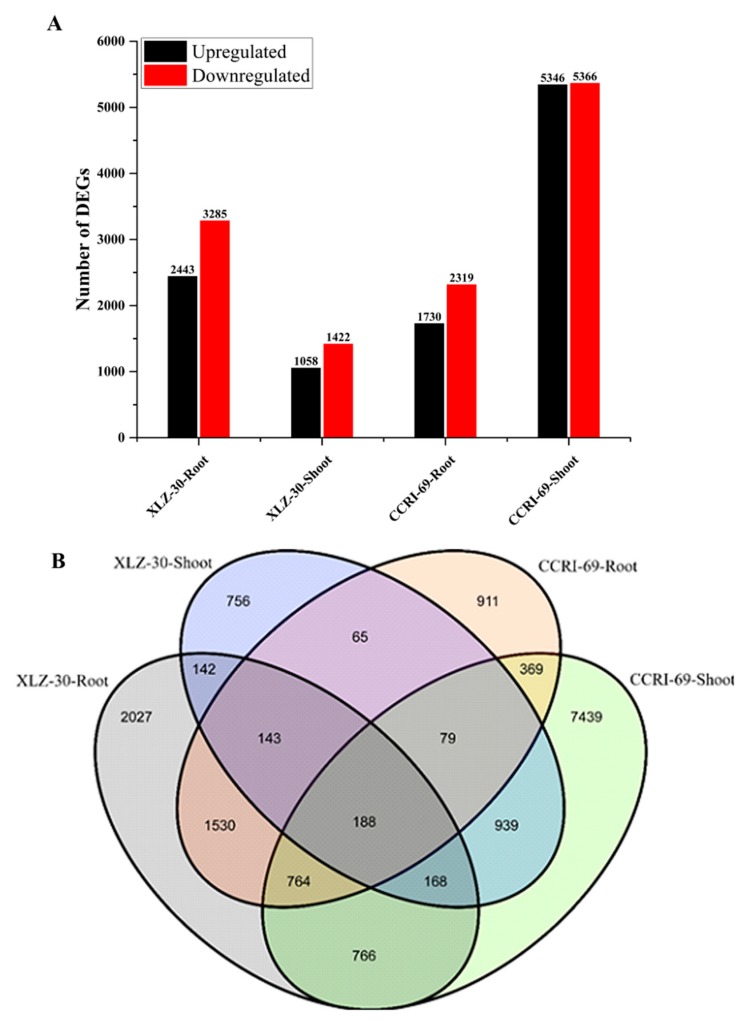
Differentially expressed genes (DEGs) of cotton genotypes at given time points during the nitrogen resupply treatments. (**A**) Number of DEGs identified by pairwise comparisons between nitrogen starvation and resupply treatments. Upregulated (black) and downregulated (red) genes were quantified. Based on DESeq software, genes with *p*-value < 0.05 were considered as significantly differential expression, and a 2-fold variance was used to identify the genes differentially expressed between the two libraries. (**B**) Venn map of DEGs among different combinations of comparisons at given time points. XLZ-30-Roots (0 h vs. 6 h), XLZ-30-Shoot (0 h vs. 6 h), CCRI-69-Root (0 h vs. 6 h), and CCRI-69-Shoot (0 h vs. 6 h) indicate cotton genotypes resupplied with nitrogen being treated with nitrogen free solution for 5 days.

To obtain the differentially expressed genes (DEGs) in the roots and shoots of cotton genotypes, we constructed four libraries under nitrogen starvation and resupply conditions. The DEGs were identified from pairwise comparisons of the four libraries, XLZ-30-Root (0 h vs. 6 h), XLZ-30-Shoot (0 h vs. 6 h), CCRI-69-Root (0 h vs. 6 h), and CCRI-69-Shoot (0 h vs. 6 h). A total of 5728 DEGs were identified in XLZ-30-Roots (0 h vs. 6 h), with 2443 upregulated and 3285 downregulated genes. In XLZ-30-Shoot (0 h vs. 6 h) the total number of DEGs was 2480 with 1058 upregulated and 1422 downregulated genes. Furthermore, in CCRI-69-Root (0 h vs. 6 h) a total of 4049 DEGs were identified with 1730 upregulated and 2319 downregulated genes. In case of CCRI-69-Shoot (0 h vs. 6 h), the total number of DEGs was 10,712, with 5346 upregulated and 5366 downregulated genes (Figure 1A). The highest number of DEGs was observed in CCRI-69-Shoot (0 h vs. 6 h), followed by XLZ-30-Roots (0 h vs. 6 h), CCRI-69-Root (0 h vs. 6 h), and XLZ-30-Shoot (0 h vs. 6 h) (Figure 1A). 

To identify common and unique DEGs in response to nitrogen starvation and resupply treatments, Venn graphs were plotted. We analyzed DEGs that were transcriptionally regulated at specific time points compared with under nitrogen starvation and nitrogen resupply conditions (Figure 1B). A total of 188 common DEGs were identified among the four libraries (Figure 1B). Thus, the DEGs display obvious differences in their nitrogen responses at the transcriptional level, and these differences depended on the plant tissue and time at which nitrogen was resupplied to cotton genotypes.

### 2.3. Gene Ontology (GO) Enrichment Analysis of DEGs

**Figure 2:**
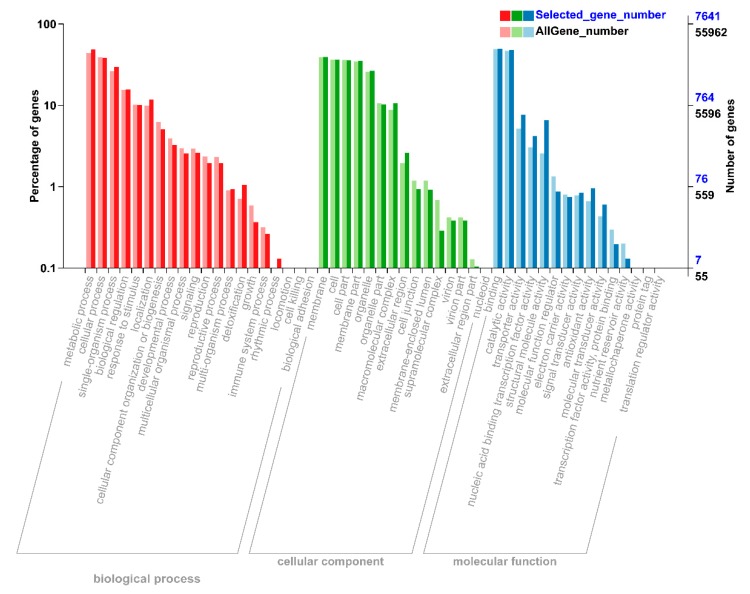
Differential annotation gene ontology (GO) annotation classification chart. The abscissa is the GO classification, the left side of the ordinate is the percentage of the number of genes, and the right side is the number of genes. This figure shows the background of the differentially expressed genes (DEGs) and the background of all genes. The DEGs were identified by pairwise comparisons between N-starvation and resupply treatments at *p*-value < 0.05. The gene enrichment of each secondary function of GO reflects the status of each secondary function in two contexts. The secondary function with obvious proportional difference indicates that the differentially expressed gene and the rich of all genes is rich.

To understand the functions of all these DEGs, we performed gene ontology (GO) analysis to classify all the DEGs into respective biological, molecular, and cellular components. According to biological function, the DEGs were mainly mapped to metabolic, cellular processes, and signal-organism (Figure 2). In the cellular component, most of the DEGs were significantly enriched in the DEGs that mapped to membrane, cell, cell part, membrane part, and organelle (Figure 2). According to molecular process, the DEGs that mapped to binding, catalytic activity, and transporter activity constituted a high proportion under N starvation and resupply treatments (Figure 2). Thus, DEGs were involved in metabolic process, signaling, nutrient reservoir activity, enzyme regulator activity, and transporter activity responding to nitrogen starvation and resupply treatments. 

### 2.4. Kyoto Encyclopedia of Genes and Genomes (KEGG) Enrichment Analysis of DEGs

**Figure 3:**
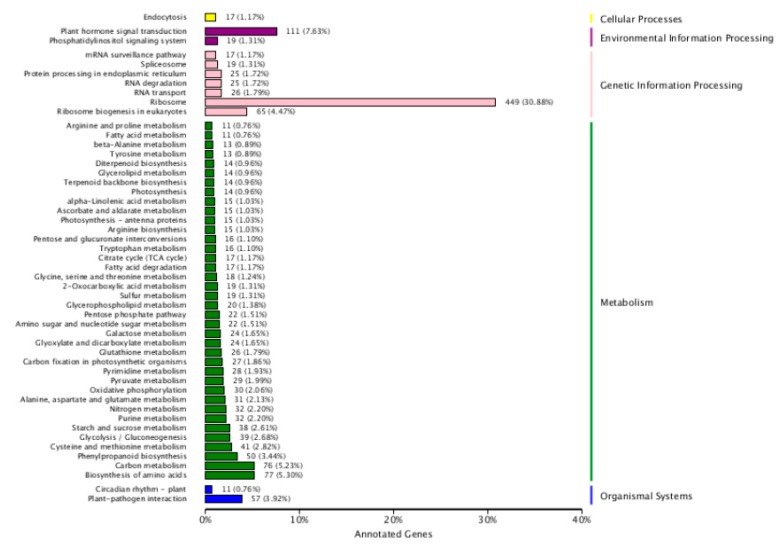
Top five Kyoto Encyclopedia of Genes and Genomes (KEGG) pathways for cotton genotypes under nitrogen starvation and resupply treatments. The ordinate is the name of the KEGG metabolic pathway, and the abscissa is the ratio of the number of genes annotated to the pathway and the number of genes on the annotation. The DEGs were identified by pairwise comparisons between N-starvation and resupply treatments at *p*-value < 0.05.

Based on the enrichment factors and Q-values, the DEGs were mapped to various metabolic pathways using a KEGG database analysis. There are five KEGG pathway categories: cellular processes, environmental information processing, genetic information processing, metabolism, and organismal systems. “Plant hormones signal transduction” was the only item enriched in environmental information. In regard to metabolism, “biosynthesis of amino acid and carbohydrate metabolism” was the most highly overrepresented, followed by “Phenylpropanoid biosynthesis” “cysteine and methionine metabolism”, “glycolysis/Gluconeogenesis”, “starch and sucrose metabolism”, “purine metabolism”, “nitrogen metabolism”, “alanine aspartate and glutamate metabolism”, and “oxidative phosphorylation”. “Ribosome” and “ribosome biogenesis in eukaryotes” were the only items enriched in genetic information processing. “Plant-pathogenic interaction” was the only item enriched in the organismal system. No enrichment was found in cellular processes (Figure 3). According to their analyses, these pathways explained the possible roles of these DEGs after nitrate starvation and resupply in the metabolism of cotton. 

### 2.5. DEGs Involved in Root Amino Acid, Carbon, and Nitrogen Metabolism

**Figure 4:**
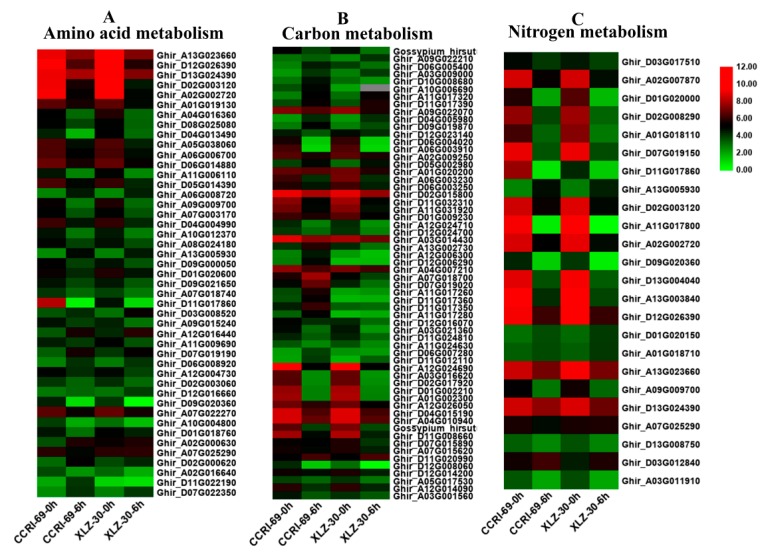
The expression patterns of genes involved in amino acid (**A**), carbon (**B**), and nitrogen (**C**) metabolism in the root of two cotton genotypes. The heat map represents the relative expression levels of genes based on Fragments Per Kilobase Million (FPKM) values using RNA sequencing (RNA-seq) data.

In this study, DEGs involved in amino acid, carbon, and nitrogen metabolic pathways were identified according to KEGG pathway analysis. Many genes involved in amino acid metabolism were differentially expressed under nitrogen starvation and resupply condition. According to the metabolic pathway analysis of these DEGs, there were 85 DEGs involved in 10 different amino acid metabolic pathways in the roots of cotton genotypes in response to N resupply (Figure 4A and Table S1). Among the genotypes, XLZ-30 had a total of 40 DEGs with 7 upregulated and 33 downregulated. While in CCRI-69, a total of 45 DEGs were found with 11 upregulated and 34 downregulated associated with amino acid metabolism (Table S1). The expression patterns of these DEGs in CCRI-69 differed from those in XLZ-30, in which transcript levels of most DEGs were increased both in CCRI-69 and XLZ-30 at 0 h (N starvation), and remained being enhanced in CCRI-69 but declined in XLZ-30 for some DEGs at 6 h. In addition, 11 DEGs were upregulated only in CCRI-69, and 7 DEGs were upregulated in XLZ-30. Three and two upregulated DEGs encoding alanine, aspartate, and glutamate metabolism were found in CCRI-69 and XLZ-30, respectively (Table S1). Moreover, two upregulated DEGs associated with glycine, serine, and threonine metabolism were found only in CCRI-69. Interestingly, most of the DEGs were downregulated in response to N resupply in both genotypes.

Carbon included various sugars, in particular, sucrose, glucose, and organic acids, which provide both the energy and the carbon skeletons for nitrogen assimilation during amino acid biosynthesis [25]. Carbon metabolism might play important roles under different nitrogen supplies. According to the metabolic pathway analysis of the DEGs, there were 106 DEGs involved in 11 different carbon metabolic pathways in roots under N starvation and resupply condition. Compared with N starvation, 9 DEGs were upregulated in XLZ-30 upon N resupply, while in CCRI-69, 21 upregulated DEGs were found encoding various pathways responsible for carbon metabolism. The transcript levels of the most DEGs were increased both in CCRI-69 and XLZ-30 under N starvation and remained being enhanced in CCRI-69 but declined in XLZ-30 for glyoxylate and dicarboxylate metabolism and glycolysis/gluconeogenesis and carbon fixation upon N resupply (Figure 4B and Table S1). Interestingly, six DEGs were upregulated, associated with glyoxylate and dicarboxylate metabolism only in CCRI-69 (Figure 4B and Table S1). Moreover, four upregulated DEGs associated with glycolysis/gluconeogenesis were found in CCRI-69 and only one in XLZ-30. Two DEGs related to carbon fixation were upregulated only in CCRI-69 after N resupply treatment. Most of the DEGs related to glycolysis/gluconeogenesis and pentose phosphate pathway were downregulated in response to N resupply in both genotypes; however, the extent of downregulation was greater in XLZ-30.

Many genes involved in nitrogen metabolism were differentially expressed under N starvation and resupply condition. In the current study, 42 DEGs involved in seven different nitrogen metabolic pathways in the roots of cotton genotypes were identified under N starvation and resupply condition. Of these DEGs, two DEGs related to glutamine synthetase and one related to cytochrome b5 were upregulated in both CCRI-69 and XLZ-30, and all of the other DEGs were downregulated in roots under N resupply condition (Figure 4C and Table S1). 

### 2.6. DEGs Involved in Shoot Amino Acid, Carbon, and Nitrogen Metabolism

**Figure 5:**
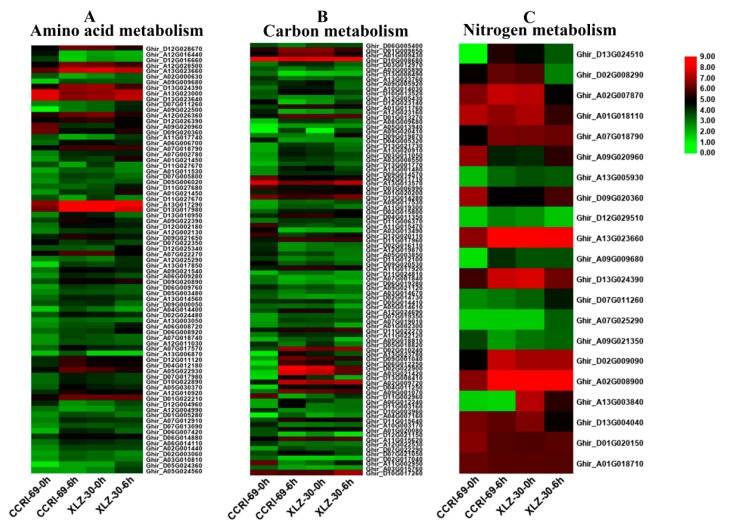
The expression patterns of genes involved in amino acid (**A**), carbon (**B**) and nitrogen (**C**) metabolism in shoot of two cotton genotypes. The heat map represents the relative expression levels of genes based on FPKM values using RNA sequencing (RNA-seq) data.

After uptake, nitrate is assimilated into ammonium and then into the amino acids glutamine, glutamate, asparagine, and aspartate, which served as important nitrogen carriers in plants. Of these metabolic pathways, DEGs that were involved in amino acid metabolism were still to be further analyzed. When subjected to nitrogen starvation and resupply treatment, there were 110 DEGs (77 upregulated and 33 downregulated) involved in 17 different amino acid metabolism processes in the shoots of cotton genotypes (Figure 5A and Table S2). The number of upregulated DEGs were more in CCRI-69 (56) than XLZ-30 (21), while the downregulated DEGs were more in XLZ-30 (23) than CCRI-69 (10). Of these DEGs, the highest number of upregulated DEGs were found for “phenylalanine, tyrosine, and tryptophan biosynthesis”, “cysteine and methionine metabolism”, and “alanine, aspartate, and glutamate metabolism”, each with eight DEGs in CCRI-69; however, zero, two, and four DEGs related to “phenylalanine, tyrosine, and tryptophan biosynthesis”, “cysteine and methionine metabolism” and “alanine, aspartate, and glutamate metabolism”, respectively, were upregulated in XLZ-30. Seven upregulated DEGs associated with “glycine, serine, and threonine metabolism” were upregulated in CCRI-69; however, only one DEG was found upregulated in XLZ-30. In addition, six and five upregulated DEGs were found for “arginine biosynthesis” and “valine, leucine and isoleucine biosynthesis”, respectively, in CCRI-69, while in XLZ-30, five DEGs related to “arginine biosynthesis” and two DEGs related to “valine, leucine and isoleucine biosynthesis” were found upregulated (Figure 5A and Table S2). 

When exposed to N resupply after 5 days of N starvation, there were 141 DEGs involved in 12 different carbon metabolic pathways in the shoots of cotton genotypes (Figure 5B and Table S2). The abundance of these DEGs was increased under N resupply treatment, whereas, the expression patterns of these DEGs in CCRI-69 differed from those in XLZ-30, in which transcript levels of most DEGs were increased in CCRI-69 than XLZ-30 at 6 h of N resupply. The highest number of upregulated DEGs were found in the shoot of CCRI-69 (54) as compared to XLZ-30 (19). Of these DEGs, 19 and 12 DEGs related to starch and sucrose metabolism and glycolysis/gluconeogenesis pathways, respectively, were significantly upregulated in CCRI-69; however, eight starch and sucrose metabolism and five glycolysis/gluconeogenesis were upregulated in XLZ-30 under N starvation and resupply treatment. Four upregulated DEGs related to pentose phosphate pathway were identified in the shoots of nitrogen-efficient genotype CCRI-69 and two DEGs were downregulated in XLZ-30. Five and four DEGs related to pentose phosphate pathway and galactose metabolism, respectively, were significantly upregulated in CCRI-69; however, three galactose metabolism encoding DEGs were upregulated, and no DEGs related to pentose phosphate pathway were upregulated in XLZ-30. There were four upregulated pentose and glucoronate interconversions-related DEGs in CCRI-69, but only one in XLZ-30. In addition, three Phenylpropanoid biosynthesis-related DEGs were upregulated in CCRI-69 and only one in XLZ-30.

Two DEGs, each related to carbon fixation and fructose and mannose metabolism, were upregulated in CCRI-69; however, only one DEG related to carbon fixation and one DEG related to fructose and mannose metabolism were upregulated in XLZ-30. These genes might be important for carbon metabolism in contrasting nitrogen-efficient cotton genotypes in response to N starvation and resupply treatments (Figure 5B and Table S2).

The relative expression levels of DEGs responsible for nitrogen metabolism in CCRI-69 exceeded those of XLZ-30 (Figure 5C and Table S2). These specific candidate genes were considered to be more important in improving N assimilation in response to N resupply in cotton. Two DEGs were related to nitrate reductase, two DEGs to asparagine synthetase, and one DEG to cytochrome b5. These DEGs were upregulated in the shoot of CCRI-69 and downregulated in N-inefficient genotype XLZ-30 under N resupply treatment (Figure 5C and Table S2). These DEGs might be essential for high nitrogen-use efficiency as found only in N-efficient genotype. Moreover, four DEGs associated with nitrate reductase were downregulated in XLZ-30, which may be the cause of lower N assimilation under N starvation and resupply treatments. 

### 2.7. Coexpression Networks Reveal a Differential Regulatory Network of Amino Acid, Carbon, and Nitrogen Metabolism under N Starvation and N Resupply

**Figure 6:**
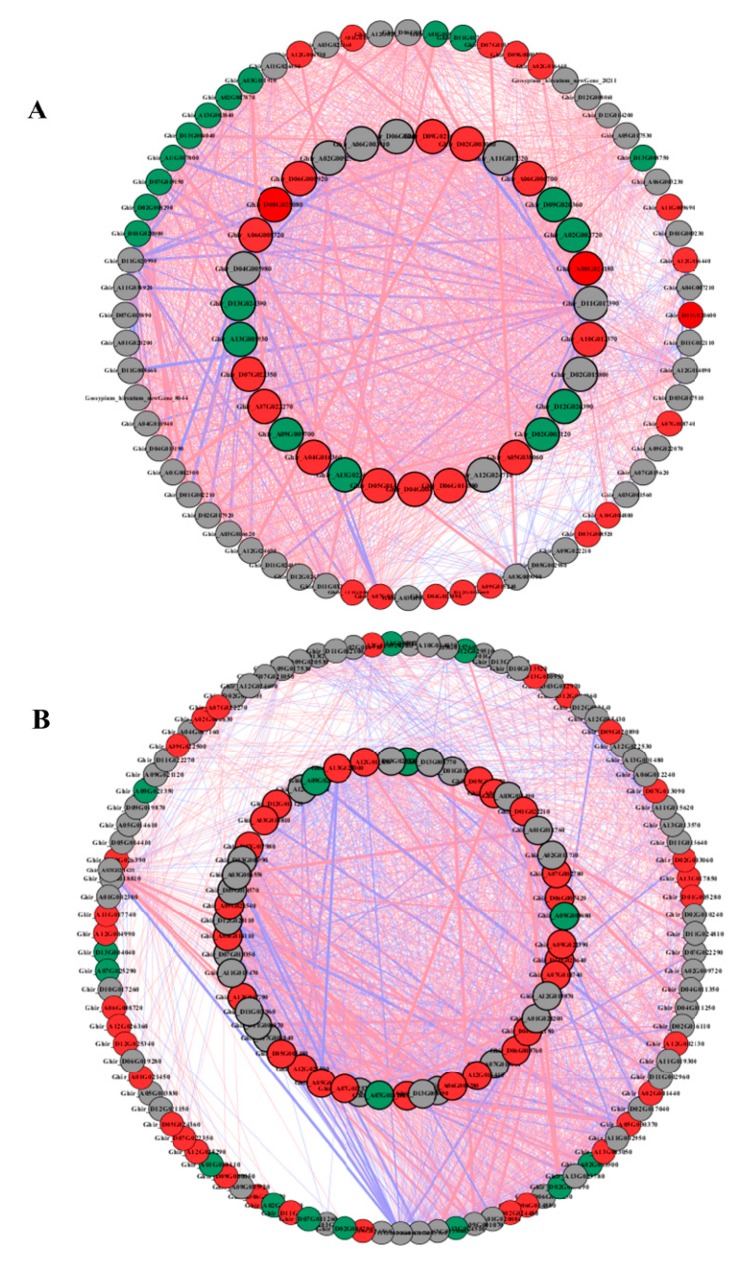
The Pearson correlation network reveals the regulatory mechanisms of amino acid, carbon, and nitrogen metabolism. (**A**) Coexpression network of genes in roots; (**B**) coexpression network of genes in shoots. Different colors of nodes represent amino acid (red), carbon (gray), and nitrogen (green). Red edges represent positive correlations and blue edges represent negative correlations. The thickness of each edge represents the value of the correlation coefficient for each correlated pair. Hub genes are highlighted with thick and black edging. The coexpression networks were made with *p* > 0.95 as the threshold.

To understand the regulatory network of amino acid, carbon, and nitrogen metabolism under N starvation and resupply, we selected 45, 61, and 20 DEGs from the root and 77, 92, and 18 DEGs from the shoots of cotton genotypes related to amino acid, carbon, and nitrogen metabolism for Pearson correlation analysis. We plotted all pairs of regulatory relationships using a threshold of a Pearson correlation coefficient greater than 0.95 (Figure 6). The visualization in Cytoscape revealed that a total of 135 nodes were connected in the network with 1698 edges in the roots, and 189 nodes were connected with in the network with 3969 edges in the shoots under N starvation and resupply treatments. According to the edges greater than 40, we obtained 25, 9, and 7 genes in the roots for regulating amino acid, carbon, and nitrogen metabolism (Figure 6A and Table S3). In case of shoot 29, 6, and 5, genes related to amino acid, carbon, and nitrogen metabolism were identified under N starvation and resupply treatments (Figure 6B and Table S4). In the root, 1704 genes showed a positive correlation, and 323 pairs were negatively correlated. Similarly, in the shoot, 2497 genes showed positive correlation and one thousand seven hundred and one genes were negatively correlated. 

### 2.8. Activities of the Key N Assimilation Enzymes

**Figure 7:**
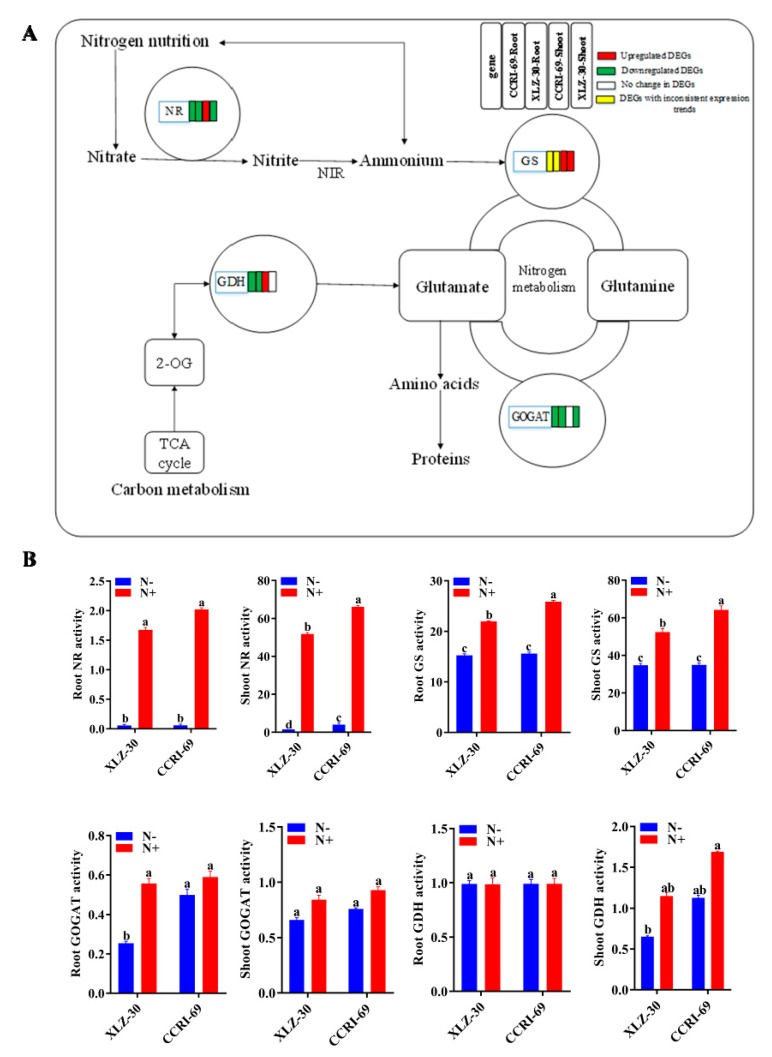
The DEGs of N metabolism pathway (**A**) and activities of key N assimilating enzymes (**B**) in the roots and shoots of CCRI-69 and XLZ-30 under N starvation and resupply treatments. The DEGs of the N metabolism pathway in the roots and shoots of CCRI-69 and XLZ-30 under N starvation and resupply treatments. The rectangles behind the gene, which were tagged with red, green, white, or yellow color, represent the upregulated DEGs, downregulated DEGs, unchanged DEGs, or the DEGs with inconsistent expression trend, respectively. N^−^, N starvation and N+, mean N resupply. NR, nitrate reductase (µg g^−1^ FW h^−1^); GS, glutamine synthetase (µmol g^−1^ FW h^−1^); GOGAT, glutamate synthase (U mg^−1^ protein); GDH, glutamate dehydrogenase (U mg^−1^ protein). Bars with different letters indicate significant difference (*p* < 0.05).

Of the four key enzymes studied for their specific activity, nitrate reductase (NR) and glutamine synthetase (GS) showed reduced activity in roots and shoots of both genotypes under N starvation (Figure 7). For glutamate dehydrogenase (GDH) and glutamate synthase (GOGAT) activities, XLZ-30 showed a reduction under N starvation, while no significant difference was noted for CCRI-69. Thus, in cases where enzymes showed differential responses between the genotypes, CCRI-69 showed increased or no change in enzymatic activities. Interestingly, genotype differences for NR and GS existed even under N resupply conditions. However, these genotype differences disappeared under N starvation for GS and root NR but not for shoot NR, which remained higher for CCRI-69. Among all the enzymes analyzed, NR was the most severely affected in both genotypes under N starvation (Figure 7).

### 2.9. Validation of the Expression Patterns of Selected DEGs by qRT-PCR

**Figure 8:**
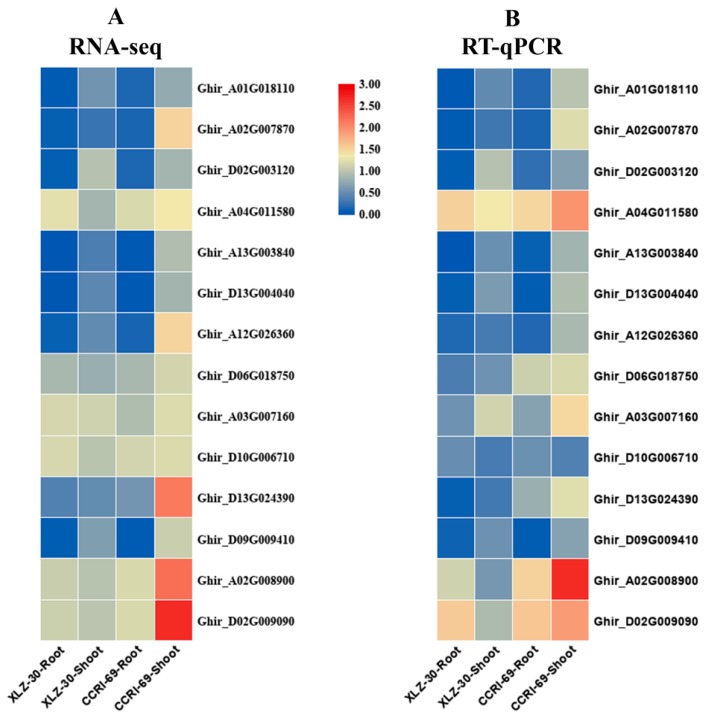
Validation of 14 differentially expressed genes (DEGs) related to nitrogen metabolism identified from transcriptome analysis by q-PCR. (**A**) RNA-seq based expression profiling; (**B**) q-PCR based expression profiling. The results were given as mean of three technical and biological replicates and expressed as fold change in mRNA relative expression.

To confirm the reliability of RNA-Seq data, qRT-PCR analysis was performed to quantify the transcript levels of fourteen genes related to nitrogen metabolism using gene-specific premiers (Table S5). The transcript data from the RNA-Seq and qRT-PCR analysis were compared using fold change measurements. The qPCR results of fourteen genes were consistent with that of RNA-Seq analysis apart from a few quantitative variations, confirming the reliability of the RNA-Seq data used in the study (Figure 8). 

## 3. Discussion

It is well known that nitrogen is very important for normal growth and development, and its deficiency is a major issue in global crop production. The development of crop cultivars with high NUE is one of the best solutions to address this issue. Studies have shown that a large variation existed among the species and genotypes within the species for NUE. Similarly, we also found a large genotypic variation among cotton genotypes for nitrogen-use efficiency based on various morphophysiological and biochemical traits [9]. However, the actual mechanisms responsible for carbon and nitrogen metabolism and subsequently for NUE in cotton have not been studied so far. Therefore, in this study, transcriptional alterations in N-efficient and -inefficient cotton genotypes CCRI-69 and XLZ-30 were investigated to better understand the mechanisms of carbon and nitrogen metabolism in response to N starvation and resupply treatments. Our data indicated that N starvation and resupply significantly affected the expression of numerous genes. The discussion section will focus on which key genes/pathways play an important role in improving amino acid, carbon and nitrogen metabolism in cotton genotypes, which can be further used for improving nitrogen use efficiency (NUE).

### 3.1. Abundance of Transcripts in the Major Pathways Related to Amino Acid, Carbon, and Nitrogen Metabolism

The result of RNA-Seq data showed a clear variation in the differentially expressed genes (DEGs) responsible for amino acid, carbon, and nitrogen metabolism in cotton genotypes. When subjected to the N resupply treatment after five days of N starvation, most of the DEGs related to amino acid, carbon, and nitrogen metabolism were downregulated in the roots of both cotton genotypes (Figure 4 and Table S1). In a similar trend, more than 2000 DEGs involved in carbon metabolism have been identified in Arabidopsis in response to nitrogen supply [42]. The downregulation of the DEGs related to major pathways were “arginine biosynthesis” and “cysteine and methionine metabolism” in amino acid metabolism, “glycolysis/gluconeogenesis” and “pentose phosphate pathway” in carbon metabolism, and “nitrate reductase”, “glutamine synthetase”, and “asparagine synthetase” in nitrogen metabolism in the roots of both cotton genotypes. The reduction in both the pentose phosphate pathway (PPP) and nitrate reduction processes upon resupply of nitrogen are in line with the previous results in rice [43]. Similarly, a dramatic downregulation of DEGs that participated in nitrate reduction and carbon assimilation pathways were found in the roots of pear [44]. In other species, the assimilation pathways were induced, while carbon assimilation was suppressed, during short-time or long-time nitrate starvation [45]. The downregulation of these DEGs may, therefore, be the components involved in the feedback response to N starvation and resupply; however, further research is needed to evaluate this hypothesis. 

It has been noted that the transcription of six DEGs related to “glyoxylate and dicarboxylate metabolisms” were upregulated in the roots of CCRI-69 but not in XLZ-30 (Figure 4 and Table S1). The glyoxylate cycle is important for the biosynthesis of carbohydrates from fatty acids, and as a result, sucrose accumulation occurs in the plant tissues. Therefore, we may assume that the enhanced glyoxylate and dicarboxylate expression in the roots of the CCRI-69 may stimulate the biosynthesis of carbohydrates from fatty acid and carbon partitioning in favor of sucrose accumulation for counteracting N starvation upon resupply of nitrogen. The enhanced sucrose accumulation in CCRI-69 might be the reason for its strong root system as observed in the previous experiments [9]. In addition, 19 DEGs related to starch and sucrose metabolism were upregulated in the shoots of CCRI-69, indicating that CCRI-69 had a great potential to enhance carbon metabolism as compared to XLZ-30. Similar findings were observed in drought and low nitrogen stresses in other studies [46,47,48]. 

In the current study, 110, 141, and 28 DEGs related to amino acid, carbon, and nitrogen metabolism, respectively, were identified in the shoots of cotton genotypes under nitrogen starvation and resupply treatments (Figure 5 and Table S2). However, the significant changes in these metabolic pathways, including “phenylalanine, tyrosine, and tryptophan biosynthesis”, “cysteine and methionine metabolism”, “alanine, aspartate, and glutamate metabolism” and “glycine, serine, and threonine metabolism” in amino acid metabolism and “starch and sucrose metabolism” and “glycolysis/gluconeogenesis” in carbon metabolism were restored and upregulated mainly in the shoot of CCRI-69 when nitrogen was resupplied within 0 to 6 h. Moreover, four DEGs related to pentose phosphate pathway (PPP) were upregulated in the shoot of CCRI-69 but not in XLZ-30. This shows that the conversion of 6-phosphogluconate to ribulose-5-phosphate, with the generation of NADPH, occurs in CCRI-69 but not in XLZ-30 in response to N starvation and resupply treatment. As one of the primary end products of the pentose phosphate pathway, NADPH is necessary for fatty acid synthesis and is needed in response to oxidative stress produced during N starvation condition [46]. The production of NADPH in the pentose phosphate pathway also provides the main reducing power for various synthesis reactions of cells, such as nitrate reduction [49]. Apart from that, it also helps to maintain the reduced state of glutathione (GSH) by serving as cosubstrate for glutathione reductases that reduce oxidized glutathione [46]. Similarly, a significant alteration in gene expression levels, including those involved with primary and secondary metabolism and hormone responses, were found within 30 min of resupplying nitrate to nitrate-starved Arabidopsis seedlings [37]. 

Previous studies have also shown that PPP is closely related to the synthesis of aromatic amino acids [48]. In the current study, we also found that the PPP changes were consistent with the content of aromatic amino acids in the shoots of CCRI-69. As eight DEGs related to “phenylalanine, tyrosine, and tryptophan biosynthesis” and one DEG related to “histidine biosynthesis” were upregulated in the shoots of CCRI-69 but not in XLZ-30. Phenylalanine (PAL) acts as an interphase between primary and secondary metabolism and is the rate limiting enzyme in Phenylpropanoid synthesis [50]. In addition, PAL plays a vital role in plant development and also helps in plant stress response. Therefore, its biosynthesis is stimulated in plants exposed to biotic and abiotic stresses including nitrogen starvation stress [50] and is, therefore, considered as one of the main markers of environmental stress [51]. Possibly, the response to N starvation could be partly altered through the regulation of PAL. As mentioned earlier, eight DEGs encoding PAL were found only in the shoots of CCRI-69 under N resupply, suggesting that the Phenylpropanoid metabolism pathway mediated by PAL regulation may confer to the genotypic difference in N use efficiency under 5-d-starved cotton plants. In a similar pattern, the low nitrogen tolerance in the barley genotype was attributed to enhanced Phenylpropanoid metabolism under limited N supply [46]. Similarly, the upregulation of DEGs, related to the Phenylpropanoid metabolism pathway in the shoot of CCRI-69, may contribute to its high N use efficiency.

### 3.2. Nitrogen Metabolic Networks in Response to Nitrogen Starvation and Resupply Treatments

Nitrogen assimilation is a fundamental biological process in plants, which is very energy consuming [52]. The energy cost is particularly larger when nitrate is used as a major N source [51]. After uptake, the nitrogen is converted into glutamine and glutamate in the roots or mainly in the shoots, which is then used to synthesized amino acids and other nitrogenous compounds [53]. N assimilation in the roots required energy and carbon skeleton produced in leaves during respiration [27]. Thus, N assimilation site is important due to its effect on the energy budget in the plants. In the current study, a downregulation in the DEGs related to N assimilation was found under N-starvation and resupply treatments. This might be helpful to keep energy supply more efficient than sucrose translocation to the roots. On the other hand, ammonium and nitrate are translocated to the shoots for assimilation. Thus, the use of little energy for N assimilation may contribute to the high NUE of CCRI-69. 

After resupplying nitrogen for 6 h to cotton genotypes that had undergone nitrogen starvation for 5 d, metabolites from the primary nitrogen metabolism pathways, such as “nitrate reductase”, “glutamate dehydrogenase”, “asparagine synthetase” and “cytochrome b5”, which were initially involved in the nitrogen assimilation process, were upregulated only in the shoot of CCRI-69 but not in XLZ-30 (Figure 5C and Table S2). This indicated that nitrogen-assimilation-related DEGs in CCRI-69 were rapidly stimulated within 6 h in response to N resupply, which is consistent with the previous studies where gene expression occurred within a short period of time after N resupplied [44]. In Arabidopsis and rice, the genes related to nitrate signaling and metabolism were altered within 30 min of N supply [54,55]. Similarly, a rapid increase in the expression profile of genes related to N metabolism was observed in nitrate-starved tomato plants within 24 h after nitrate application [40]. In another study, thousands of genes responsible for N assimilation were induced or repressed in Arabidopsis roots within 20 min after nitrate application [38]. Thus, it was concluded that the pathways related to N assimilation are sensitive to N supply and regulated in a coordinated manner to balance nutrients and signals. 

Besides gene expression, the activities of enzymes involved in N metabolism are also very important. Previous studies showed that NR activities in tobacco and Arabidopsis mutants altered, and could not assimilate, nitrate while maintaining the ability to respond to the available nitrate [56,57]. In the current study, large genotypic variations were also observed among the key enzymes regulating N metabolism. NR and GS play important roles in N assimilation for plants grown under low N availability [58]. Ye et al. [59] found higher NR and GS activities in a higher N-efficient barley genotype. A similar result was also observed in the present study, where N-efficient genotype (CCRI-69) had higher enzymatic activities, indicating its greater potential for N metabolism (Figure 7). Inconsistent with our results, high N-based enzymatic activities were found in Arabidopsis [60] and N-efficient *Brassica napus* genotypes [59]. The variations in N-based enzymatic activities among the genotypes may be associated with differences in regulation of N-related transcripts in the roots [61]. Similarly, the N-metabolism-related genes were more active in the roots and shoots of CCRI-69, which might lead to the high N uptake and assimilation. Additionally, the high-N efficiency of CCRI-69 may result from a well-coordinated system of N uptake and assimilation, resulting in low ammonia levels in the tissues [62]. Moreover, the high NUE of CCRI-69 can be explained by the significantly increased enzymatic activities in the roots and shoots at the physiological level and the significant upregulation of N metabolism genes at the molecular level. In a similar pattern, the increase in N enzymatic activities followed by the abundance of their transcripts was found in low-N-tolerant sugarcane genotype [63]. Thus, the increased expression of genes and enzymatic activities related to N metabolism suggested that CCRI-69 shoots recovered from the state of nitrogen starvation within 6 h of resupplying nitrogen. 

### 3.3. Molecular Mechanism and Regulation of Carbon and Nitrogen Metabolism

The identification of the differences in the molecular mechanism and regulations networks between CCRI-69 and XLZ-30 under nitrogen starvation and resupply can provide a new approach for understanding the complex regulatory mechanisms of carbon and nitrogen metabolism [64,65]. Moreover, maintaining an appropriate balance between carbon and nitrogen metabolites in the cell, which was referred to as “carbon/nitrogen balance” was also important for the regulation of plant growth, development, and yield [22,27]. In a good agreement with the carbon/nitrogen balance requirements, when exposed to N starvation and resupply treatments during 0 to 6 h period, 42 and 34 DEGs involved in carbon metabolism were downregulated in the roots of XLZ-30 and CCRI-69, respectively (Figure 4B). In the shoots, 9 and 21 upregulated DEGs that participated in the carbon metabolism were found in XLZ-30 and CCRI-69, respectively, in response to N starvation and resupply treatments (Figure 5B). 

Another phenomenon could be interpreted by a demand-driven adaptation/balancing hypothesis: under the starvation condition, the insufficient nitrogen acquisition was assumed to occur; thus, nitrogen became the limiting substrate in incorporating glutamate to generate glutamine and subsequently downstream amino acid and carbon metabolism. As a result, under N starvation, the demand for the energy and the carbon skeletons for nitrogen assimilation was scaled down; therefore, more proteins involved in carbon metabolism downregulated to coordinate amino acid metabolism. To survive under N starvation, some genes related to the alleviation of the detrimental effect were abundantly expressed, thus resulting in enhanced metabolic activities in the plant. 

### 3.4. Hypothesis

**Figure 9:**
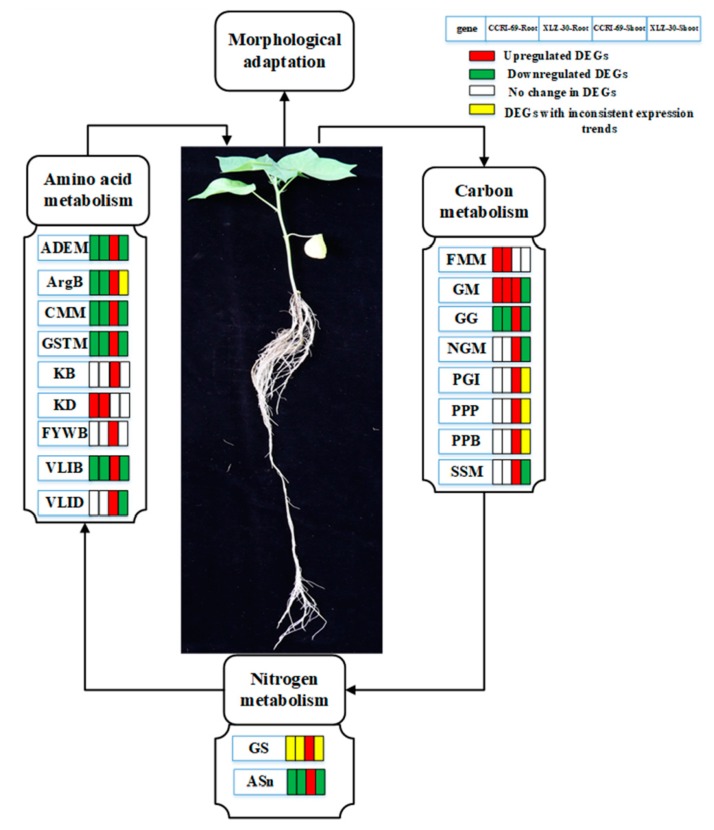
Schematic model representing the hub genes in different metabolic pathways related to amino acid, carbon, and nitrogen metabolism in the roots and shoots of cotton under N starvation and resupply responses. The rectangles behind the genes, which were tagged with red, green, white, and yellow colors, represent the upregulated DEGs, downregulated DEGs, unchanged DEGs, and the DEGs with inconsistent expression trend, respectively. The Hub genes related to major pathways in amino acid metabolism were ADEM (alanine aspartate, glutamate metabolism), ArgB (Arginine biosynthesis), CMM (cysteine and methionine metabolism), GSTM (glycine, serine, and threonine metabolism), KB (lysine biosynthesis), KD (lysine degradation), FYWB (phenylalanine, tyrosine, and tryptophan biosynthesis), VLIB (valine, leucine, isoleucine biosynthesis), and VLID (valine, lysine, and isoleucine degradation). The hub genes involved in carbon metabolism were FMM (fructose and mannose metabolism), GM (galactose metabolism), GG (glycolysis/gluconeogenesis), NGM (N-Glycan metabolism), PGI (Pentose and glucuronate interconversions), PPP (pentose phosphate pathway), PPB (Phenylpropanoid biosynthesis), and SSM (starch and sucrose metabolism) and that for nitrogen metabolism were GS (glutamine synthetase) and ASn (asparagine synthetase).

Taking the identified hub genes into consideration, we hypothesized that nitrogen resupplied after five days’ starvation can effectively uptake by the roots and be converted into amino acids through nitrogen metabolism. The amino acids formed are then translocated into the shoots and assimilated into organic nitrogen in the leaves via N metabolism. Carbohydrates produced in the leaves are converted into the roots to promote plant growth and development. During this process, some key genes/pathways responsible for amino acid, carbon, and nitrogen metabolism changed, which may affect the corresponding physiological and biochemical processes in both roots and shoots (Figure 9). Thus, genotypes that can efficiently uptake the available nitrogen and may assimilate it into amino acid and other N compounds are considered to be N-efficient. 

## 4. Materials and Methods

### 4.1. Plant cultivation and Nitrogen Treatment

The experiment was carried out in a greenhouse at the Cotton Research Institute of the Chinese Academy of Agriculture Sciences, Anyang, China. Healthy seeds of two cotton genotypes (CCRI-69, N-efficient, and XLZ-30, N-inefficient) were placed in a mixture of sand and vermiculate for one week in a germinator. After the full opening of two cotyledons, seedlings with uniform height were selected and transplanted into 7 L containers in the growth chamber (16/8 h light/dark cycle, 28 °C temperature, 60% relative humidity). After transplanting, the seedlings were supplied with half-strength during the first week, followed by a full-strength, Hoagland solution as reported by Iqbal et al. [66]. At three-leaves stage, seedlings were exposed to nitrogen-free nutrients solution (N starvation) for five days. After five days, the seedlings were resupplied with 2 mM Ca(NO_3_)_2_ which contained 4 mM NO_3_^−^ as normal nitrogen, and subsequently, the root and shoot samples were taken at 0 and 6 h after treatment. 

### 4.2. Measurement of Key Enzymes Activities in N Metabolism

The roots and shoots samples were collected at 0 h and 6 h after N resupply for the measurement of NR, GS, GOGAT, and GDH activities.

Nitrate reductase (NR) activity was measured according to Silveira et al. [67] and expressed as μg NO_2_^−^ h^−1^ g^−1^ fresh weight (FW). About 0.2 g of fresh samples were grounded in liquid nitrogen. Following the addition of 2 mL extraction solution [9], it was then centrifuged at 8000 rpm for 10 min at 4 °C. The supernatant was then mixed with 1.6 mL of a mixture containing 1.2 mL of 0.1 M KNO_3_ phosphate buffer and 0.4 mL of 2.0 mg·mL^−1^ NADH solution, while the control was kept as NADH-free and kept in a water bath at 30 °C for 30 min. Then, 1.0 mL of 1% p-aminobenzene sulfonic acid and 0.2% α-naphthylamine were added and kept for 20 min till color development. Finally, the NR activity was measured at a wavelength of 540 nm using a spectrophotometer. 

The GS enzyme activity was assessed by grinding fresh samples (0.2 g) on ice with an extraction containing 2.0 mL of an extract [68] and centrifuged at 15,000 rpm for 20 min at 4 °C. After centrifugation, 0.7 mL of supernatant was mixed with 1.6 mL of 0.1 M Tris–HCL buffer (pH 7.4, 80.0 mM MgSO_4_, 20.0 mM sodium glutamate, 20.0 mM cysteine, 2.0 mM EDTA, and containing 80.0 mM HONH3Cl) and 0.7 mL of 40.0 mM ATP solution. The mixture was placed in water bath at 25 °C for 30 min, to which 1.0 mL of a chromogenic reagent (0.2 M trichloroacetic acid, 0.37 M FeCl_3_, and 0.6 M HCl) was added, incubated for 15 min, and centrifuged at 5000 rpm for 10 min at 25 °C; then, the supernatant was collected, and the absorbance was measured at a wavelength of 540 nm. The reaction mixture of 1.6 mL of 0.1 M Tris–HCl solution (pH 7.4, not containing 80.0 mM HONH_3_Cl) was added as control.

The activities of GOGAT and GDH were measured at a wavelength of 340 nm using a spectrophotometer [69]. The enzyme extraction was similar to GS activity. After enzyme extraction, 100 mM K^+^-phosphate, containing 0.1% (*v*/*v*) 2-mercaptoethanol, was used for the measurement. The reaction solution of GOGAT was 2.5 mM α-ketoglutarate, 100 μM NADH, 10.0 mM L-glutamine, and 1.0 mM aminooxyacetate, and that of GDH was 2.5 mM α-ketoglutarate, 100.0 μM NADH, and 100.0 mM (NH_4_)_2_SO_4_. The final values were calculated as the oxidation of 1 noml of NADH per min. 

### 4.3. RNA-Seq Sampling, RNA Extraction, and mRNA-Seq Library Construction for Illumina Sequencing

For RNA-Seq sampling, the samples were taken at 0 h and 6 h after resupplying of nitrogen (4 mM NO_3_^−^). Roots and shoots of four seedlings for each genotype were collected and mixed at each time point. All samples (total 24, 2 genotypes (CCRI-69, N-efficient, and XLZ-30 N-inefficient) × 2 plant tissues (root and shoot) × 2-time points (0 h and 6 h) × 3 biological replications) were prepared for further RNA-Seq analysis.

Total RNA was extracted from both the tissues of both genotypes using a miRNeasy mini kit (QIAGEN, Hilden, Germany). The degradation and contamination of RNA were monitored on 1% agarose gels, and the purity of RNA was checked using a NanoPhotometer spectrophotometer (Implen, Health Care Facilities & Svcs, La Baya Drive West Lake, CA, USA). RNA concentrations were measured using a Qubit RNA Assay Kit in a Qubit 2.0 Fluorometer (Life Technologies, Carlsbad, CA, USA). The RNA integrity was evaluated using the RNA Nano 6000 Assay Kit of the Agilent Bioanalyzer 2100 system (Agilent Technologies, Stevens Creek Blvd, Santa Clara, CA, USA).

Sequencing libraries were generated according to the manufacturer’s instructions (Illumina, San Diego, CA, USA) using NEB Next Ultra RNA Library Prep Kit for Illumina (New England Biolabs, NEB, USA) following the manufacturer’s instructions, and index codes were added to be able to attribute sequences to each sample. PCR products were purified (AMPure XP system). Fragmentation buffer was added to cleave mRNA into small fragments. These fragments were then used as templates to synthesize the first stranded cDNA with RNase H and DNA polymerase I. The cDNA was used to construct a paired-end library using Genomic Sample Prep Kit (Illumina). The fragments with a desirable length were purified with QIAquick PCR (Qiagen) Extraction Kit, end-repaired, and linked with sequencing adapters [70]. The unsuitable fragments were removed through AMPureXP beads, and the sequencing library was constructed with PCR. After being checked with Pico green staining and fluoro-spectrophotometry and quantified with Agilent 2100, the multiplexed DNA libraries were mixed by equal volume with normalized 10 nM concentration. The sequencing library was then sequenced with the Illumina HiSeq platform (Shanghai Personal Biotechnology Co., Ltd., Shanghai, China).

### 4.4. Data Filtering, Mapping of Reads, and Functional Annotation

The raw reads were generated through the Illumina data processing pipeline (version 1.8, Boston, MA, USA). For further analysis, the clean data were obtained by removing low-quality bases, empty reads, and adaptor sequences at the 3′ end from the raw reads. Meanwhile, the Q30, GC contents, and sequence duplication level of the clean data were calculated. *Gossypium hirsutum* TM-1 genome [71] and gene model annotation files (CottonGen database, http://www.cottongen.org) were used for compiling a reference genome for clean-read alignments. 

For gene expression analysis, fragments per kilobase of exon per million fragments mapped reads (FPKM) was calculated at expression level [72]. The difference in expression (three biological replicates per time point) was analyzed using the DESeq R package (1.10.1) [73]. An FDR (false discovery rate) was set as 0.05 for the threshold of DEGs [74].

Then we performed GO (http://geneontology.org/) and KEGG (http://www.genome.jp/kegg/) function enrichment analysis to the differentially expressed genes (DEGs). GO annotation and KEGG analysis for the DEGs were performed using the Blast2GO program [75] and the similar steps as reported by [45]. The annotation result of GOs distribution associated with DEGs was categorized with respect to the biological process, molecular function, and cellular component [76]. The KEGG maps, which contained the EC numbers and enzymatic functions in many metabolic pathways were performed using the online KEGG Automatic Annotation Server (KAAS) (http://www.genome.jp/tools/kaas/).

### 4.5. Coexpression Network Analysis of Genes Related to Amino Acid, Carbon and Nitrogen Metabolism

Coexpression network analyses were conducted to determine the relationships among genes responsible for amino acid, carbon, and nitrogen metabolism [77]. According to the method of Zhang et al. [46], Cytoscape software (version 3.3.0, Cytoscape, San Diego, CA, USA) was used to construct a coexpression regulation network of the genes responsible for amino acid, carbon, and nitrogen metabolism. The coexpression network map was made with *p* > 0.95 as the threshold [78]. The hub genes within the network were identified according to the topological coefficient of each node with degree >40.

### 4.6. Validation of RNA-Seq Analysis by qRT-PCR

In total, 14 candidate genes associated with nitrogen metabolism were selected to determine the accuracy of DEG-based gene expression as assessed by qRT-PCR performed on an ABI System (7500). The RNAs of the shoot and root tissues for qRT-PCR were the same from Section 2.3. of the materials and methods section, and DNase I was used in all RNAs to avoid genomic contamination. About 1 μg sample of isolated RNA was used to synthesize the first-strand cDNA using the PrimeScript™ RT Master Mix (Perfect Real Time, TaKaRa, Japan) according to the manufacturer’s protocol. The primers specific to the 14 DEGs and the housekeeping histone3.3 (AT5G09810) were designed using Primer Premier 5.0 (Premier Biosoft, San Francisco, CA, USA). The gene-specific primers were designed using primer-blast (http:/www.ncbi.nlm.nih. gov/tools/primer-blast/), and all the primers are presented in Table S3. Three biological and three technical repeats were processed for the qRT-PCR assays, which were performed in 20 μL reaction mixture containing 2 μL cDNA, and 10 μL LightCycler 480 SYBRGREEN I Master Mix (Perfect Real Time, TaKaRa). All reactions were run as duplicates in 96-well plates. The PCR reactions were performed under the following conditions: preincubation at 95 °C for 30 s, then 40 cycles of 95 °C for 5 s, and 60 °C for 34 s. In addition, the expression levels were calculated using the 2^−ΔCt^ method for each sample. 

## 5. Conclusions

The identification of DEG transcripts in plants would reveal the genetic mechanism of NUE. Here, the results of RNA-Seq analysis demonstrated that there was a dramatic difference at the transcriptional level between the two cotton genotypes in response to N starvation and resupply. In addition, we observed that cotton genotypes experience a large difference in response to N starvation and resupply, of which the changes in amino acid, carbon, and nitrogen metabolism were the most obvious. Accordingly, a hypothetical model was developed for the mechanism of amino acid, carbon, and nitrogen metabolism to improve NUE (Figure 9). We deduced that efficient N absorption and assimilation by roots and more energy-producing by the shoots/leaves may contribute to high NUE. Moreover, coexpression network analysis was used to obtain 75 and 33 hub genes in the shoots and roots of cotton genotypes, respectively. This study not only provides insights into the molecular mechanisms of cotton response to N resupply but may be of significance in uncovering the as-yet hub genes for amino acid, carbon, and nitrogen metabolism, benefiting the breeding of N-efficient cotton genotypes. 

## Figures and Tables

**Table 1:** Summary of RNA-seq data and reads mapping of cotton genotypes under nitrogen starvation and resupply conditions.

Sample	Total Reads	Mapped Reads	Uniquely Mapped Reads	Clean Reads	Multiple Map Reads	GC (%)	Q30 (%)
T1	58,570,080	53,610,649 (91.37%)	51,091,336 (87.10%)	29,285,040	2,519,313 (4.31%)	45.19	95.22
T2	45,719,979	44,065,333 (96.38%)	41,797,242 (91.42%)	22,859,989	2,268,090 (4.96%)	44.99	95.66
T3	46,445,903	42,395,883 (91.28%)	40,346,255 (86.87%)	23,222,951	2,049,628 (4.41%)	45.02	95.44
T4	46,768,972	44,190,679 (94.60%)	41,918,645 (89.75%)	23,584,486	2,275,367 (4.84%)	45.12	95.44
T5	44,412,336	58,095,133 (93.23%)	39,466,925 (88.80%)	22,206,156	1,961,540 (4.43%)	44.88	95.42
T6	43,632,818	39,893,880 (91.41%)	37,631,996 (86.55%)	21,816,409	2,261,883 (5.03%)	45.90	95.71
T7	45,474,475	40,764,984 (89.65%)	38,554,429 (84.79%)	22,737,237	2,210,555 (4.86%)	45.06	95.70
T8	47,407,099	44,979,691 (94.88%)	42,692,137 (90.05%)	23,703,549	2,287,554 (4.83%)	45.17	95.50

T1; XLZ-30-0h-Root, T2; XLZ-30-0h-Shoot, T3; XLZ-30-6h-Root, T4; XLZ-30-6h-Shoot, T5; CCRI-69-0h-Root, T6; CCRI-69-0h-Shoot, T7; CCRI-69-6h-Root, T8; CCRI-69-6h-Shoot.

## Supplementary Materials

Supplementary materials can be found at https://www.mdpi.com/1422-0067/21/4/1500/s1.
