# Supplementary material for: Transcriptome Analysis Reveals Differences in Key Genes and Pathways Regulating Carbon and Nitrogen Metabolism in Cotton Genotypes under N Starvation and Resupply
Source: Int J Mol Sci. 2020 Feb 22;21(4):1500. doi: 10.3390/ijms21041500 (PMC7073098; doi:10.3390/ijms21041500)
Supplement: Supplementary file 1 [file ijms-21-01500-s001.pdf]

**Supplementary Table S1.** Number of up- and down-regulated DEGs responsible for amino acid, carbon and nitrogen metabolism in the roots of contrasting nitrogen efficient cotton genotypes in response to N starvation and resupply.

| | **XLZ-30** | | **CCRI-69** | |
|---|---|---|---|---|
| **Amino acid metabolism** | **Up** | **Down** | **Up** | **Down** |
| Alanine, aspartate and glutamate metabolism | 2 | 4 | 3 | 5 |
| Arginine biosynthesis | 2 | 10 | 2 | 10 |
| Cysteine and methionine metabolism | 1 | 6 | 2 | 6 |
| Glutathione metabolism | 0 | 1 | 0 | 1 |
| Glycine, serine and threonine metabolism | 0 | 5 | 2 | 5 |
| Histidine metabolism | 0 | 2 | 0 | 2 |
| Lysine degradation | 2 | 0 | 2 | 0 |
| Monobactam biosynthesis | 0 | 1 | 0 | 1 |
| Pyrimidine metabolism | 0 | 1 | 0 | 1 |
| Valine, leucine and isoleucine biosynthesis | 0 | 3 | 0 | 3 |
| Carbon metabolism | | | | |
| Amino sugar and nucleotide sugar metabolism | 1 | 1 | 1 | 0 |
| Carbon fixation | 0 | 1 | 2 | 0 |
| Glyoxylate and dicarboxylate metabolism | 0 | 2 | 6 | 0 |
| Starch and sucrose metabolism | 0 | 5 | 0 | 4 |
| Glycolysis / Gluconeogenesis | 1 | 15 | 4 | 12 |
| Pentose phosphate pathway | 0 | 12 | 0 | 11 |
| Fructose and mannose metabolism | 2 | 1 | 2 | 1 |
| Galactose metabolism | 2 | 1 | 2 | 1 |
| Fatty acid elongation | 1 | 0 | 1 | 0 |
| Pentose and glucuronate interconversions | 1 | 4 | 2 | 4 |
| Phenylpropanoid biosynthesis | 1 | 0 | 1 | 1 |
| Nitrogen metabolism | | | | |
| Nitrate reductase | 0 | 4 | 0 | 4 |
| Glutamine synthetase | 2 | 6 | 2 | 4 |
| Nitrite reductase | 0 | 2 | 0 | 2 |
| Glutamate synthase | 0 | 1 | 0 | 1 |
| Glutamate dehydrogenase | 0 | 1 | 0 | 1 |
| Asparagine synthetase | 0 | 3 | 0 | 4 |
| cytochrome b5 | 1 | 2 | 1 | 1 |

**Supplementary Table S2.** Number of up- and down-regulated DEGs responsible for amino acid, carbon and nitrogen metabolism in the shoots of contrasting nitrogen efficient cotton genotypes in response to N starvation and resupply.

| | XLZ-30 | | CCRI-69 | |
|---|---|---|---|---|
| **Amino acid metabolism** | **Up** | **Down** | **Up** | **Down** |
| Alanine, aspartate and glutamate metabolism | 4 | 6 | 8 | 3 |
| Arginine and proline metabolism | 4 | 1 | 2 | 2 |
| Arginine biosynthesis | 5 | 2 | 6 | 2 |
| Cysteine and methionine metabolism | 2 | 7 | 8 | 1 |
| Glycine, serine and threonine metabolism | 1 | 3 | 7 | 0 |
| Histidine metabolism | 0 | 0 | 1 | 0 |
| Lysine biosynthesis | 0 | 1 | 2 | 0 |
| Lysine degradation | 2 | 0 | 1 | 0 |
| Monobactam biosynthesis | 0 | 0 | 2 | 0 |
| Phenylalanine metabolism | 0 | 0 | 2 | 0 |
| Phenylalanine, tyrosine and tryptophan biosynthesis | 0 | 1 | 8 | 0 |
| Pyrimidine metabolism | 0 | 0 | 1 | 0 |
| Purine metabolism | 1 | 0 | 0 | 1 |
| Synthesis and degradation of ketone bodies | 0 | 0 | 2 | 0 |
| Taurine and hypotaurine metabolism | 0 | 1 | 1 | 0 |
| Valine, leucine and isoleucine biosynthesis | 2 | 1 | 5 | 0 |
| Amino sugar and nucleotide sugar metabolism | 0 | 0 | 0 | 1 |
| Carbon metabolism | | | | |
| Carbon fixation | 1 | 2 | 2 | 1 |
| Cutin, suberine and wax biosynthesis | 0 | 2 | 1 | 0 |
| Cyanoamino acid metabolism | 0 | 1 | 1 | 2 |
| Fructose and mannose metabolism | 0 | 2 | 2 | 1 |
| Galactose metabolism | 3 | 2 | 5 | 2 |
| Glycolysis / Gluconeogenesis | 5 | 5 | 12 | 5 |
| N-Glycan biosynthesis | 0 | 0 | 1 | 0 |
| Oxidative phosphorylation | 0 | 0 | 0 | 1 |
| Pentose and glucuronate interconversions | 1 | 5 | 4 | 12 |
| Pentose phosphate pathway | 0 | 2 | 4 | 0 |
| Phenylpropanoid biosynthesis | 1 | 2 | 3 | 1 |
| Starch and sucrose metabolism | 8 | 11 | 19 | 9 |
| Nitrogen metabolism | | | | |
| Nitrate reductase | 0 | 4 | 2 | 1 |
| Glutamine synthetase | 2 | 1 | 2 | 1 |
| Nitrite reductase | 0 | 2 | 0 | 0 |
| Glutamate synthase | 0 | 2 | 0 | 0 |
| Glutamate dehydrogenase | 0 | 0 | 1 | 0 |
| Asparagine synthetase | 2 | 2 | 3 | 0 |
| cytochrome b5 | 0 | 0 | 2 | 1 |

**Supplementary Table S3.** Hub genes up and down-regulated in the root of contrasting N-efficient cotton genotypes in response to N starvation and resupply.

| Category | Gene ID | CCRI-69 | XLZ-30 |
|---|---|---|---|
| Amino acid | Ghir_D13G024390 | Downregulated | Downregulated |
| Amino acid | Ghir_A13G023660 | Downregulated | Downregulated |
| Amino acid | Ghir_A09G009700 | Downregulated | Downregulated |
| Amino acid | Ghir_A13G023660 | Downregulated | Downregulated |
| Amino acid | Ghir_D02G003120 | Downregulated | Downregulated |
| Amino acid | Ghir_A06G006700 | Downregulated | Downregulated |
| Amino acid | Ghir_A13G005930 | Upregulated | Upregulated |
| Amino acid | Ghir_D09G020360 | Downregulated | Downregulated |
| Amino acid | Ghir_A04G016360 | Downregulated | Downregulated |
| Amino acid | Ghir_D05G014390 | Downregulated | Downregulated |
| Amino acid | Ghir_A02G002720 | Downregulated | Downregulated |
| Amino acid | Ghir_A13G005930 | Upregulated | Upregulated |
| Amino acid | Ghir_D09G020360 | Downregulated | Downregulated |
| Amino acid | Ghir_A05G038060 | Downregulated | Downregulated |
| Amino acid | Ghir_D04G004990 | Downregulated | Downregulated |
| Amino acid | Ghir_D09G021650 | Downregulated | Downregulated |
| Amino acid | Ghir_A07G022270 | Downregulated | Downregulated |
| Amino acid | Ghir_D07G022350 | Upregulated | Upregulated |
| Amino acid | Ghir_D08G025080 | Downregulated | Downregulated |
| Amino acid | Ghir_A08G024180 | Downregulated | Downregulated |
| Amino acid | Ghir_D06G014880 | Downregulated | Downregulated |
| Amino acid | Ghir_A10G012370 | Downregulated | Downregulated |
| Amino acid | Ghir_A06G008720 | Upregulated | Upregulated |
| Amino acid | Ghir_D02G003060 | Downregulated | Downregulated |
| Amino acid | Ghir_D06G008920 | Upregulated | Upregulated |
| Nitrogen | Ghir_D12G026390 | Downregulated | Downregulated |
| Nitrogen | Ghir_D02G003120 | Downregulated | Downregulated |
| Nitrogen | Ghir_A02G002720 | Downregulated | Downregulated |
| Nitrogen | Ghir_A13G005930 | Upregulated | Upregulated |
| Nitrogen | Ghir_D09G020360 | Downregulated | Downregulated |
| Nitrogen | Ghir_A13G023660 | Downregulated | Downregulated |
| Nitrogen | Ghir_A09G009700 | Downregulated | Downregulated |
| Nitrogen | Ghir_D13G024390 | Downregulated | Downregulated |
| Carbon | Ghir_D11G017390 | Upregulated | Upregulated |
| Carbon | Ghir_A11G017320 | Upregulated | Upregulated |
| Carbon | Ghir_D04G005980 | Upregulated | Upregulated |
| Carbon | Ghir_D06G004020 | Downregulated | Downregulated |
| Carbon | Ghir_A06G003910 | Downregulated | Downregulated |
| Carbon | Ghir_A02G009250 | Downregulated | Downregulated |
| Carbon | Ghir_D02G015800 | Downregulated | Downregulated |

| | | | |
|---|---|---|---|
| Carbon | Ghir_A12G024710 | Downregulated | Downregulated |

**Supplementary Table S4.** Hub genes up and down-regulated in the shoot of contrasting N-efficient cotton genotypes in response to N starvation and resupply.

| Category | Gene ID | CCRI-69 | XLZ-30 |
|---|---|---|---|
| Amino acid | Ghir_A09G021540 | Upregulated | Downregulated |
| Amino acid | Ghir_A13G017290 | Upregulated | Downregulated |
| Amino acid | Ghir_D13G017980 | Upregulated | Downregulated |
| Amino acid | Ghir_D12G002180 | Upregulated | Downregulated |
| Amino acid | Ghir_A13G023000 | Downregulated | Upregulated |
| Amino acid | Ghir_A06G009280 | Upregulated | Downregulated |
| Amino acid | Ghir_D06G009760 | Upregulated | Downregulated |
| Amino acid | Ghir_A06G006700 | Upregulated | Downregulated |
| Amino acid | Ghir_A12G011030 | Upregulated | Downregulated |
| Amino acid | Ghir_D05G003480 | Upregulated | Downregulated |
| Amino acid | Ghir_A12G028500 | Upregulated | Downregulated |
| Amino acid | Ghir_A13G014560 | Upregulated | Downregulated |
| Amino acid | Ghir_A09G009680 | Upregulated | No change |
| Amino acid | Ghir_D09G020360 | Downregulated | Upregulated |
| Amino acid | Ghir_A07G018740 | Upregulated | Downregulated |
| Amino acid | Ghir_D12G028670 | Upregulated | Downregulated |
| Amino acid | Ghir_A07G018790 | Upregulated | Downregulated |
| Amino acid | Ghir_A07G012910 | Upregulated | Downregulated |
| Amino acid | Ghir_A06G014110 | Upregulated | Downregulated |
| Amino acid | Ghir_A01G011530 | Upregulated | Downregulated |
| Amino acid | Ghir_D07G017980 | Upregulated | Upregulated |
| Amino acid | Ghir_A09G020960 | Downregulated | Upregulated |
| Amino acid | Ghir_A12G016440 | Downregulated | No change |
| Amino acid | Ghir_D13G023640 | Downregulated | Upregulated |
| Amino acid | Ghir_D06G007420 | Upregulated | Downregulated |
| Amino acid | Ghir_D10G022890 | Upregulated | Downregulated |
| Amino acid | Ghir_D01G022210 | Upregulated | Upregulated |
| Amino acid | Ghir_D04G012180 | Upregulated | No change |
| Amino acid | Ghir_A05G022930 | Upregulated | Downregulated |
| Amino acid | Ghir_A07G002780 | Upregulated | No change |
| Amino acid | Ghir_D07G005800 | Upregulated | Downregulated |
| Amino acid | Ghir_D12G011120 | Upregulated | Downregulated |
| Amino acid | Ghir_A07G017570 | Upregulated | Downregulated |
| Amino acid | Ghir_A04G014400 | Upregulated | Downregulated |
| Amino acid | Ghir_D05G006020 | Upregulated | No change |
| Amino acid | Ghir_D13G024390 | Upregulated | Downregulated |
| Amino acid | Ghir_A03G010810 | Upregulated | Downregulated |
| Amino acid | Ghir_A09G022390 | Upregulated | Downregulated |

| | | | |
|---|---|---|---|
| Amino acid | Ghir_D09G021650 | Upregulated | Downregulated |
| carbon | Ghir_D10G008680 | Downregulated | Upregulated |
| carbon | Ghir_A01G011760 | Upregulated | Downregulated |
| carbon | Ghir_A08G009680 | Upregulated | Downregulated |
| carbon | Ghir_D04G005320 | Downregulated | No change |
| carbon | Ghir_A03G008550 | Downregulated | Downregulated |
| carbon | Ghir_D13G008490 | Downregulated | No change |
| carbon | Ghir_D01G013270 | Upregulated | Downregulated |
| carbon | Ghir_D03G011050 | Upregulated | Downregulated |
| carbon | Ghir_A01G020200 | Upregulated | Downregulated |
| carbon | Ghir_D09G014570 | Upregulated | Downregulated |
| carbon | Ghir_A02G011710 | Downregulated | Upregulated |
| carbon | Ghir_A12G023160 | Downregulated | Upregulated |
| carbon | Ghir_D11G006370 | Upregulated | Downregulated |
| carbon | Ghir_D11G017960 | Upregulated | Downregulated |
| carbon | Ghir_A13G023760 | Downregulated | Upregulated |
| carbon | Ghir_A05G013940 | No change | No change |
| carbon | Ghir_A13G020910 | Upregulated | Downregulated |
| carbon | Ghir_D13G001770 | Upregulated | Downregulated |
| carbon | Ghir_A11G017920 | Upregulated | Downregulated |
| carbon | Ghir_D03G006990 | Downregulated | Upregulated |
| carbon | Ghir_A03G013490 | Downregulated | No change |
| carbon | Ghir_D12G020110 | Downregulated | Upregulated |
| carbon | Ghir_A12G019870 | Downregulated | No change |
| carbon | Ghir_A07G019010 | Upregulated | No change |
| carbon | Ghir_D13G021730 | Upregulated | Downregulated |
| carbon | Ghir_D07G019350 | Upregulated | Downregulated |
| carbon | Ghir_A07G001840 | Downregulated | No change |
| carbon | Ghir_A11G022120 | Upregulated | No change |
| carbon | Ghir_A11G015470 | Downregulated | Upregulated |
| carbon | Ghir_D09G001040 | Upregulated | Downregulated |
| Nitrogen | Ghir_D09G020360 | Downregulated | Upregulated |
| Nitrogen | Ghir_A09G009680 | Upregulated | No change |
| Nitrogen | Ghir_A13G023660 | Upregulated | Downregulated |
| Nitrogen | Ghir_A07G018790 | Upregulated | Downregulated |
| Nitrogen | Ghir_A09G020960 | Downregulated | Upregulated |
| Nitrogen | Ghir_D13G024390 | Upregulated | Downregulated |

**Supplementary Table S5.** Gene specific primers used for RT-qPCR.

| Gene ID | Gene name | Specific primer |
|---|---|---|
| Ghir_A01G018110 | NR-1F | TGGTATGGTATTCGAGCACCCA |
| Ghir_A01G018110 | NR-1R | GTCGTAGATATGACCATGGAC |
| Ghir_A02G007870 | NR-2F | TCGAAGGAGCTGAGCATTTG |

| | | |
|---|---|---|
| Ghir_A02G007870 | NR-2R | TTCGTTGTAACAATGATTCGT |
| Ghir_D02G003120 | GS-1F | GTGGAGTTCTCTGGTACTGA |
| Ghir_D02G003120 | GS-1R | AACGTATTCAGCGATGATCT |
| Ghir_A04G011580 | GS-2F | ATCGATGAGAAATGATGGTGG |
| Ghir_A04G011580 | GS-2R | GGCAGTCTCGTGAAGACCT |
| Ghir_A13G003840 | NiR-1F | GGATTGAAGTGTTCAGATCAGAG |
| Ghir_A13G003840 | NiR-1R | GTTCGTCCATGTCGTCAGCTTGG |
| Ghir_D13G004040 | NiR-2F | GTATTGAAGTGTTCAGATCAGAAG |
| Ghir_D13G004040 | NiR-2R | GTTCGTCCATGTCGTCGGCTTG |
| Ghir_A12G026360 | GOGAT-1F | ATGAGCGTTATGGGTTGTAA |
| Ghir_A12G026360 | GOGAT-1R | ACATCACCAACGAGATGCAA |
| Ghir_D06G018750 | GOGAT-2F | GTGAAGCCCAGTAACACGGA |
| Ghir_D06G018750 | GOGAT-2R | CTGAGCCATTTGATAGCTGATG |
| Ghir_A03G007160 | GDH-1F | TCAAGGTTTCATGTGGGATGAAG |
| Ghir_A03G007160 | GDH-1R | GTTCACCCCAAGTGTAAATGCGC |
| Ghir_D10G006710 | GDH-2F | TCTTGCAGAGTTGAATAAAGA |
| Ghir_D10G006710 | GDH-2R | CCATCCTCGTCAACCAAATACC |
| Ghir_D09G009410 | ASN-1F | GACCGAAAGACTGATGACTGA |
| Ghir_D09G009410 | ASN-1R | TAGTCTGCAACCTCCCTTGC |
| Ghir_D13G024390 | ASN-2F | TTGTTATTTGGCTCATCAACGC |
| Ghir_D13G024390 | ASN-2R | GTACTCCTCATACAAATGTGC |
| Ghir_A02G008900 | Cytb5-1F | GTGACAAAGTTCATGGAAGA |
| Ghir_A02G008900 | Cytb5-1R | GGAGCAAATTCTGAGCTGAC |
| Ghir_D02G009090 | Cytb5-2F | GAACGTAACAAAGTTCCTGGAA |
| Ghir_D02G009090 | Cytb5-2R | GAGCAAATTCTGAGCCGACTT |
| AT5G09810 | Gh H 3.3-F | CCTTGTGGGTCTTTTTGAA |
| AT5G09810 | Gh H 3.3-R | AACTGGATGTCCTTGGGC |
